# Airspace Dimension Assessment (AiDA) by inhaled nanoparticles: benchmarking with hyperpolarised ^129^Xe diffusion-weighted lung MRI

**DOI:** 10.1038/s41598-021-83975-7

**Published:** 2021-02-25

**Authors:** Madeleine Petersson-Sjögren, Ho-Fung Chan, Guilhem J. Collier, Graham Norquay, Lars E. Olsson, Per Wollmer, Jakob Löndahl, Jim M. Wild

**Affiliations:** 1 Division of Ergonomics and Aerosol Technology, Department of Design Sciences and NanoLund, Lund University, Lund, Sweden; 2 POLARIS, Imaging Sciences, Department of Infection, Immunity & Cardiovascular Disease, University of Sheffield, Sheffield, UK; 3 Department of Translational Medicine, Medical Radiation Physics, Lund University, Malmö, Sweden; 4 Department of Translational Medicine, Clinical Physiology, Lund University, Malmö, Sweden

**Keywords:** Diagnostic markers, Chronic obstructive pulmonary disease, Diagnostic devices, Imaging techniques and agents

## Abstract

Enlargements of distal airspaces can indicate pathological changes in the lung, but accessible and precise techniques able to measure these regions are lacking. Airspace Dimension Assessment with inhaled nanoparticles (AiDA) is a new method developed for in vivo measurement of distal airspace dimensions. The aim of this study was to benchmark the AiDA method against quantitative measurements of distal airspaces from hyperpolarised ^129^Xe diffusion-weighted (DW)-lung magnetic resonance imaging (MRI). AiDA and ^129^Xe DW-MRI measurements were performed in 23 healthy volunteers who spanned an age range of 23–70 years. The relationship between the ^129^Xe DW-MRI and AiDA metrics was tested using Spearman’s rank correlation coefficient. Significant correlations were observed between AiDA distal airspace radius (*r*_AiDA_) and mean ^129^Xe apparent diffusion coefficient (ADC) (p < 0.005), distributed diffusivity coefficient (*DDC*) (p < 0.001) and distal airspace dimension (*Lm*_D_) (p < 0.001). A mean bias of − 1.2 µm towards *r*_AiDA_ was observed between ^129^Xe *Lm*_D_ and *r*_AiDA_, indicating that *r*_AiDA_ is a measure of distal airspace dimension. The AiDA *R*_0_ intercept correlated with MRI ^129^Xe α (p = 0.02), a marker of distal airspace heterogeneity. This study demonstrates that AiDA has potential to characterize the distal airspace microstructures and may serve as an alternative method for clinical examination of the lungs.

## Introduction

Detection of microstructural changes in the distal airspaces can be crucial for clinical evaluation of early stage lung disease and longitudinal monitoring of pulmonary diseases. The standard procedures for detection of disease in the distal airspaces include spirometry, test of diffusing capacity of the lung for carbon monoxide (D_LCO_), and lung density analysis from computed tomography (CT). However, all the mentioned techniques can fail to reveal early indications of pathological changes^1,2^. Therefore, considerable disease can be present with minimal effect on standard pulmonary function tests (PFTs).

Diffusion-weighted (DW) magnetic resonance imaging (MRI) with inhaled hyperpolarised noble gases helium-3 (^3^He) or xenon-129 (^129^Xe) is an in vivo imaging method that is sensitive to changes in the distal airspaces^3–5^. The method is based on measurement of the Brownian diffusional restriction of the inhaled hyperpolarised gas atoms within the distal airspace walls. This property is used to derive the apparent diffusion coefficient (ADC), which provides 3D in vivo information on the distal airspace microstructure. In addition to ADC, theoretical models of hyperpolarised gas diffusion within the lungs, such as the stretched exponential model (SEM)^6,7^ and the cylinder airway model (CM)^8,9^, can be used to derive distal airspace dimensions, analogous to those obtained through histological analysis. These in vivo distal airspace measurements from hyperpolarised gas DW-MRI have shown good agreement with direct morphometric measurements in validation studies with lung specimens^8,10,11^. Numerous studies have used hyperpolarised gas DW-MRI to elucidate changes in distal airspaces related to smoking^12–14^, ageing^15^, lung inflation^16^, and diseases such as COPD^17,18^, asthma^19^, and idiopathic pulmonary fibrosis (IPF)^20^. However, due to the high cost and complex infrastructure required for hyperpolarised gas MRI, the technique is not typically employed in standard pulmonary function testing or screening for pulmonary disease.

Airspace Dimension Assessment (AiDA) with inhaled nanoparticles, could potentially be more cost efficient and more widely accessible than MRI with hyperpolarised gases. The method is based on measurement of the exhaled recovery of inhaled nanoparticles which deposit in the distal airspaces due to Brownian diffusion^21^. The fraction of deposited particles is directly related to the size of the airspaces, and analysis of the nanoparticle recovery yields two metrics. The first metric is an effective airspace radius (*r*_AiDA_), which is a root mean square measure of a collection of airspaces, and the second metric is the recovery at an imaginary zero-seconds breath-hold (*R*_0_). *r*_AiDA_ has been found to correlate with the extent of emphysema^22^ and proton lung tissue density as quantified by standard pulmonary structural MRI^23^. *R*_0_ significantly correlates with the carbon monoxide transfer coefficient (K_CO_) and age^24^. Further benchmarking of the technique with established methods of in vivo distal airspace assessment is required to evaluate the clinical potential of the AiDA technique.

The aim of this study was to benchmark inhaled nanoparticle measurements with AiDA against ^129^Xe DW-MRI derived ADC and distal airspace dimensions. Since both AiDA^21^ and ^129^Xe DW-MRI ^6^ use diffusion of nanoparticles and gas molecules, we hypothesise that measures from the two different techniques will correlate.

## Results

### Volunteer demographics, pulmonary function test data, DW-MRI metrics and AiDA variables

Table 1 summarises volunteer demographics, pulmonary function test results, hyperpolarised ^129^Xe DW-MRI metrics and AiDA values for the 23 volunteers.

**Table 1:** Summary of volunteer demographics and global measurements.

Characteristic	Mean ± SD	Median (IQR)	Min	Max
Age (years)	48 ± 17	54 (34)	23	70
Height (cm)	173.7 ± 9.8	175 (15)	155	191
Weight (kg)	75.5 ± 14.4	77 (24)	55	103
FEV_1_ (% pred)	96.0 ± 9.7	95.8 (10.9)	80.1	115.4
VC (% pred)	96.5 ± 12.4	94.8 (10.3)	74.7	132.4
TLC (% pred)	97.9 ± 11.6	96.8 (14.5)	80.3	128.2
RV (% pred)	102.5 ± 24.8	102.2 (27.6)	57	182
FRC (% pred)	93.3 ± 16.8	93.4 (25.0)	57.6	116.5
D_LCO_ (% pred)	102.8 ± 18.8	101.3 (23.7)	67.5	153.7
K_CO_ (% pred)	113.2 ± 16.2	112 (21.4)	85	150.3
ADC (cm^2^/s)	0.034 ± 0.004	0.034 (0.0041)	0.0263	0.0434
α (a.u.)	0.862 ± 0.015	0.863 (0.023)	0.826	0.889
*DDC* (cm^2^/s)	0.030 ± 0.004	0.030 (0.005)	0.022	0.030
*Lm*_*D*_ (µm)	281 ± 19	277 (20)	244	322
*r*_AiDA_ (µm)	279 ± 25	284 (42)	240	325
*R*_0_ (a.u.)	0.484 ± 0.132	0.51 (0.21)	0.183	0.675

Data are presented as mean ± SD, median (IQR), minimum values and maximum values.

*IQR* interquartile range, *FEV*_*1*_ forced expiratory volume in 1 s, *VC* vital capacity, *TLC* total lung capacity, *RV* residual volume, *FRC* forced respiratory capacity; *D*_*LCO*_ diffusing capacity of lung for carbon monoxide, *K*_*CO*_ carbon monoxide transfer coefficient, *ADC* apparent diffusion coefficient, *α* heterogeneity index, *DDC* distributed diffusivity coefficient, *Lm*_*D*_ mean diffusive length scale, *r*_AiDA_ distal airspace radius, *R*_*0*_ zero-second recovery.

### Benchmarking of AiDA using the DW-MRI measurements

**Figure 1:**
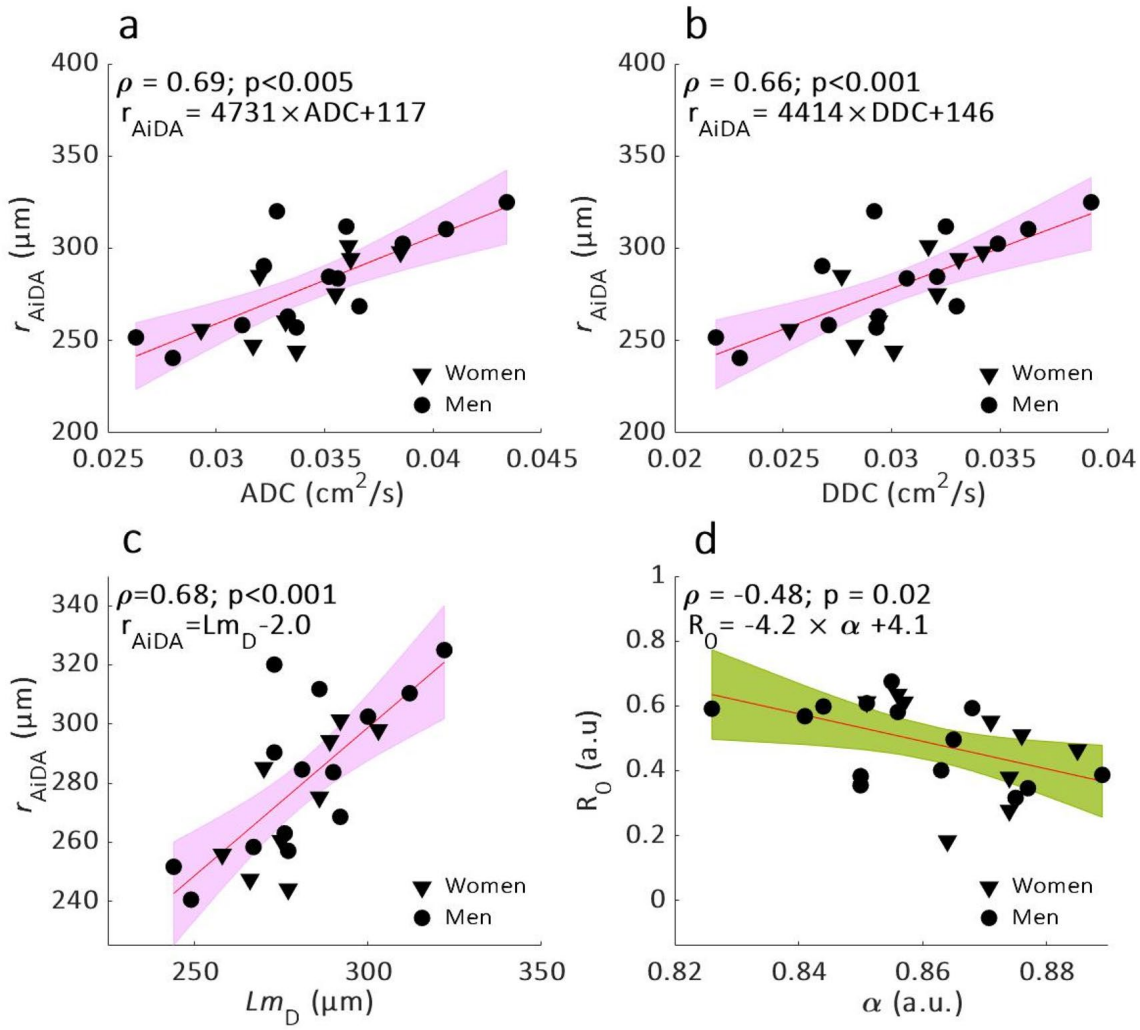
Linear regressions (with 95% confidence intervals in color) and Spearman’s correlation (ρ) of *r*_AiDA_ as a function of ^129^Xe DW-MRI metrics: (**a**) ADC, (**b**) *DDC,* (**c**) *Lm*_*D*_ and of *R*_0_ as a function of α heterogeneity index (**d**).

**Figure 2:**
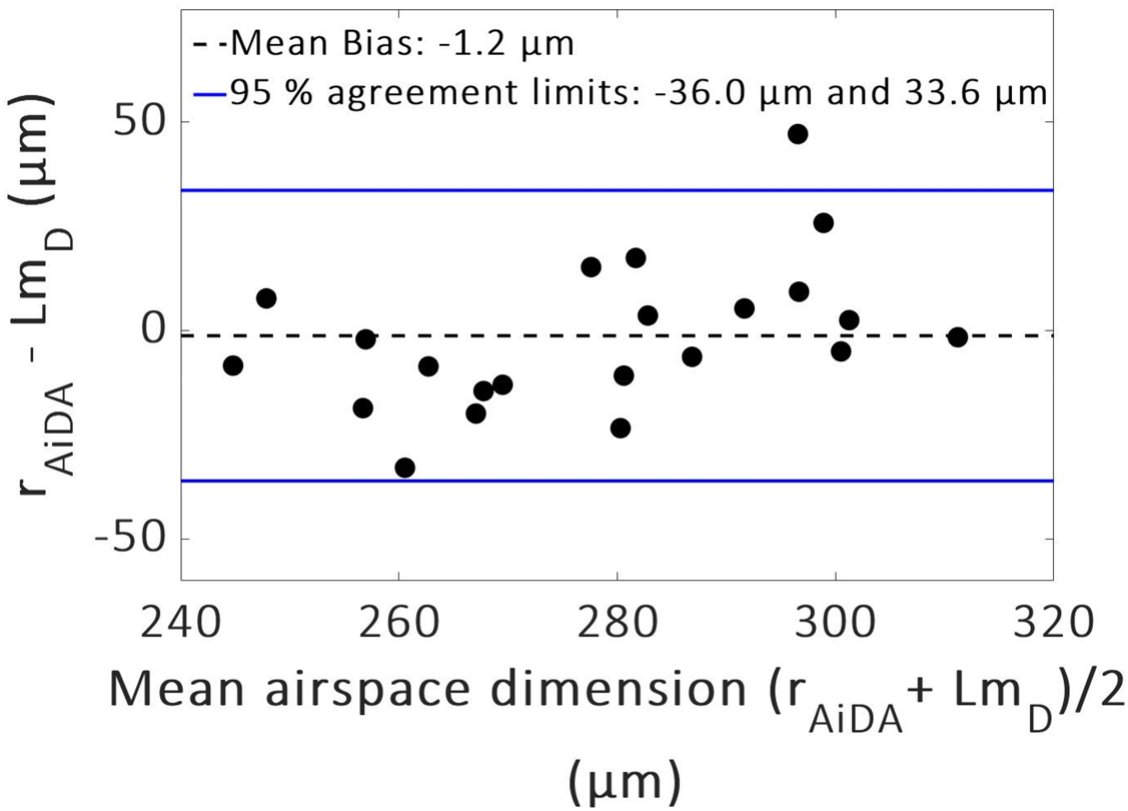
Bland–Altman plot of distal airspace radius *r*_AiDA_ and mean diffusive length scale *Lm*_*D*_. A mean bias of 1.2 µm towards r_AiDA_ and 95% agreement limits − 36.0 to 33.6 µm were found.

Statistically significant linear correlations were observed between *r*_AiDA_ and ^129^Xe DW-MRI metrics ADC (p < 0.005), *DDC* (p < 0.001) and *Lm*_*D*_ (p < 0.001) (Fig. 1a–c). The *R*_0_ intercept measurement from AiDA was significantly correlated with ^129^Xe α heterogeneity index (p = 0.02) (Fig. 1d), but not with any of the other ^129^Xe DW-MRI metrics. Bland–Altman analysis of *r*_AiDA_ and ^129^Xe *Lm*_*D*_ showed a mean bias of − 1.2 µm (95% agreement limits − 36.0 to 33.6 µm) towards *r*_AiDA_ (Fig. 2). *r*_AiDA_ and *Lm*_D_ deviated less than 0.05% for the whole group and on average 8% between subjects.

For the comparison in the Bland–Altman plot, a linear relationship of strength ρ = 0.48 was observed (p = 0.02) suggesting *r*_AiDA_ increased more than *Lm*_*D*_ with increasing airspace size. No difference was seen between men and women for the AiDA and ^129^Xe DW-MRI metric distributions.

**Figure 3:**
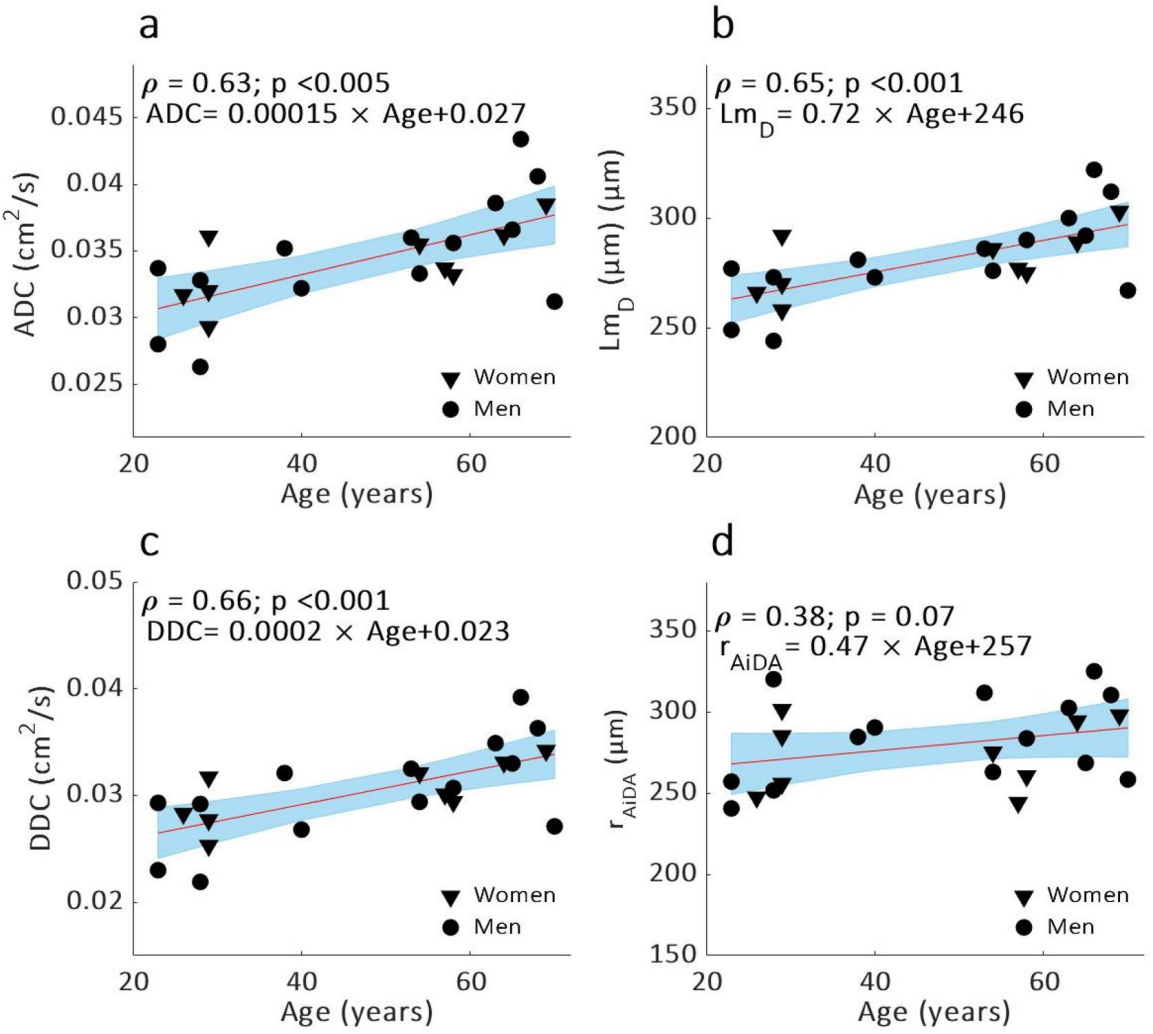
Linear regressions (with 95% confidence intervals in blue) and Spearman’s correlation (ρ) of (**a**) ADC, (**b**) *Lm*_D_, (**c**) *DDC* and (**d**) *r*_AiDA_ as a function of age.

Significant correlations were observed between volunteer age ^129^Xe DW-MRI metrics ADC (p < 0.005), *DDC* (p < 0.001) and *Lm*_*D*_ (p < 0.001) (Fig. 3a–c). A trend towards increased *r*_AiDA_ with age was observed (p = 0.07) (Fig. 3d). A positive trend between *R*_0_ and FEV_1_ was observed (p = 0.07). AiDA and ^129^Xe DW-MRI variables did not correlate significantly with any of the PFT measurements.

## Discussion

Measurements of distal airspace dimensions were acquired with AiDA and ^129^Xe DW-MRI in 23 healthy volunteers. AiDA metrics, *r*_AiDA_ and *R*_0_, corresponded to previously reported values from healthy volunteers^22–24^. ^129^Xe DW-MRI metrics of ADC, *DDC* and distal airspace dimensions (*Lm*_D_) were larger than the values previously reported for younger healthy volunteers (29 ± 4 years), but smaller than values in ex-smoker volunteers^6^.

Distal airspace radius *r*_AiDA_, significantly correlated with ^129^Xe DW-MRI metrics ADC, *DDC* and *Lm*_*D*_, which confirm to us that *r*_AiDA_ is a measure of distal airspace dimensions. Agreement between *r*_AiDA_ and ^129^Xe DW-MRI *Lm*_*D*_ was confirmed with Bland–Altman analysis where a mean bias of − 1.2 µm towards *r*_AiDA_, corresponding to 0.43% deviation from the mean measured *r*_AiDA_, was observed. Both AiDA and ^129^Xe DW-MRI measure the Brownian motion, presumably within the same distal airspace. The breath-hold times and diffusion times were optimised specifically for diffusion across small distances but assume that the particles stay within the same airway duct. The statistically significant correlation between *R*_0_ and α heterogeneity index (p = 0.02) could be indicating that *R*_0_ represents heterogeneity in the acini, which both metrics are hypothesized to measure^6,24^.

The ADC, *DDC* and *Lm*_D_ dependency on age suggests a prevalence of age-related distal airspace changes in the older healthy volunteers^25^. The significant correlations between volunteer age and ^129^Xe ADC and *Lm*_*D*_ further demonstrate the sensitivity of DW-MRI to age-related airspace changes, and matches trends previously observed with ^3^He DW-MRI^15,26^. A trend towards increasing *r*_AiDA_ with age was observed. α heterogeneity correlated with volunteer height (p < 0.001). AiDA and ^129^Xe DW-MRI metrics did not correlate with any other volunteer demographic or PFT data.

The bias and relatively wide 95% agreement limits (− 36.0 to 33.6 µm) observed in the Bland–Altman plot can be explained by the difference in acquisition method of the two techniques. AiDA is acquired over multiple breath-holds with diffusion times ranging between 5 and 15 s. In contrast, ^129^Xe DW-MRI is a single breath-hold acquisition with an 8.5 ms diffusion time. The linear trend in bias visible in the Bland Altman plot (Fig. 2) suggests that the agreement between *r*_AiDA_ and *Lm*_*D*_ changes with increasing mean airspace dimension size. This implies that the measurements from the two methods might diverge for increasing distal airspaces with an increasing bias between *r*_AiDA_ and *Lm*_*D*_ in larger airspaces. The increasing bias may be attributed to the difference between the diffusion coefficients for xenon (0.14 cm^2^/s when mixed with air in the lungs^27^ and the 50 nm nanoparticles (2 × 10^–5^ cm^2^/s^28^). Due to the much smaller mass and size of ^129^Xe-atoms they diffuse much faster than the nanoparticles. Hence, the exponential decay, from which the diffusion distance in the lung is calculated, is different for the two techniques. For ^129^Xe DW-MRI the theoretical 1D free diffusion length ($L_{1D}$) is approximately 500 µm ($L_{1D} = \sqrt {2{\Delta }D_{0} } ; {\Delta } =$ 8.5 ms, $D_{0} = 0.14$ cm^2^/s) while for 50 nm nanoparticles the corresponding displacement in one direction is approximately 200 µm (${\Delta } =$ 10 s, $D_{0} =$ 2 × 10^−5^ cm^2^/s).

While the ^129^Xe DW-MRI method provides voxel-wise regional information about the lung structure the AiDA method provides one measure of the distal airspace dimensions. Therefore, the ^129^Xe DW-MRI method can give more detailed and regional information about the structure of the distal airspaces compared to AiDA. However, as indicated by this study, the AiDA method has the potential to give a faster and more accessible, but still precise, measurement of the distal airspace dimensions, which can be of great importance when hyperpolarised lung MRI is not available. To further compare the relative sensitivity between the two methods more measurements, in particular including a large variation in distal space sizes, are needed.

Although the two methods obtained approximately similar airspace dimensions (average *r*_AiDA_ and *Lm*_*D*_ deviated less than 0.5%), there were also several significant differences in the measurement procedures for the subjects. ^129^Xe DW-MRI measurements were made at FRC + 1 L while AiDA measurements were made at TLC. Hence, the lung inflation was larger for all AiDA measurements as compared to ^129^Xe DW-MRI measurements. Previous studies have shown that measured ADC in hyperpolarised ^3^He DW-MRI increases with increasing lung inflation volumes^29^. Which means that, AiDA could be expected to have measured larger dimensions *r*_AiDA_ when compared to *Lm*_*D*_.

Distal airspace size also depends on posture and ADC decreases from the non-dependent region of the lung down to the dependent region. For hyperpolarised ^3^He DW-MRI measurements, ADC has been found to vary significantly depending on posture, and this was attributed to the compression of parenchyma, due to the lungs own weight, and the mass of the heart^30^. In this study AiDA measurements were performed in upright sitting position while the ^129^Xe DW-MRI measurements were performed in supine position. Therefore, a postural variation between whole lungs AiDA measurement and the regionally averaged ^129^Xe DW-MRI metrics is expected. For a more elaborate comparison of the two methods the breath-hold volumes could be set to be equal and breath-hold times set to correspond to the same diffusion distances. In addition, AiDA could potentially be measured with subjects in a supine posture enabling an even more efficient comparison with minimized systematic errors.

AiDA measurements have been shown to be repeatable to approximately < 2.4% when measured at different times over a period of 18 months^31^. Previous studies have shown that ^3^He and ^129^Xe ADC is highly repeatable in COPD patients with a coefficient of variation of 2.98% and 2.77% respectively, over 5 visits^32^. Similarly it has been showed that ^3^He *Lm*_D_ in patients with idiopathic pulmonary fibrosis is highly repeatable with a 0.6% difference between same-day visits^20^.

This is the first study that compares AiDA with an independent and validated non-invasive method for assessment of distal airspaces. The study includes a limited number of healthy subjects, which mainly was due to the logistics related to travel between Sweden and the UK. The subject group was thus homogenous with small variations in PFT results. However, even with the small group of volunteers, significant correlations were found between AiDA and ^129^Xe DW-MRI metrics, and interestingly these increased with age indicating age dependent changes in alveolar size or ‘aging emphysema’^25^. Ideally, a future extension of this study would include subjects with a greater range of distal airspace sizes, such as patients with emphysematous lung disease.

In conclusion, this work has compared estimates of airspace radii from inhaled nanoparticles by the AiDA method with ^129^Xe DW-MRI in a healthy volunteer cohort. The significant correlations show that the distal airspace radius of the lungs measured by AiDA (*r*_AiDA_) can be related to distal airspace microstructure dimensions as quantified by ^129^Xe DW-MRI. Quantitavely the two methods are in close agreement, with mean *r*_AiDA_ and *Lm*_*D*_ deviating < 0.5% for the whole group and on average 8% on an individual level. Further benchmarking in selected groups of patients could be used to evaluate the relative sensitivity of AiDA and ^129^Xe DW-MRI in detecting early emphysematous changes to the distal airspace microstructure.

## Methods

### Study subjects and study design

The study enrolled 23 healthy adult volunteers (14M, 9F) in the age range 23–70 years with no history of pulmonary disease, in Sweden in the spring 2019. The study was approved by the Regional Ethical Review Board in Lund, Sweden (application number 2018/659), and performed in accordance with the Declaration of Helsinki, including obtaining informed written consent from all volunteers. All volunteers underwent spirometry, body plethysmography, carbon monoxide gas transfer, and AiDA measurements at Skåne University Hospital in Malmö, Sweden. Pulmonary function tests were performed according to the European Respiratory Society guidelines^33^. All ^129^Xe DW-MRI measurements were performed at University of Sheffield, Sheffield, UK**.**

### AiDA measurements

**Figure 4:**
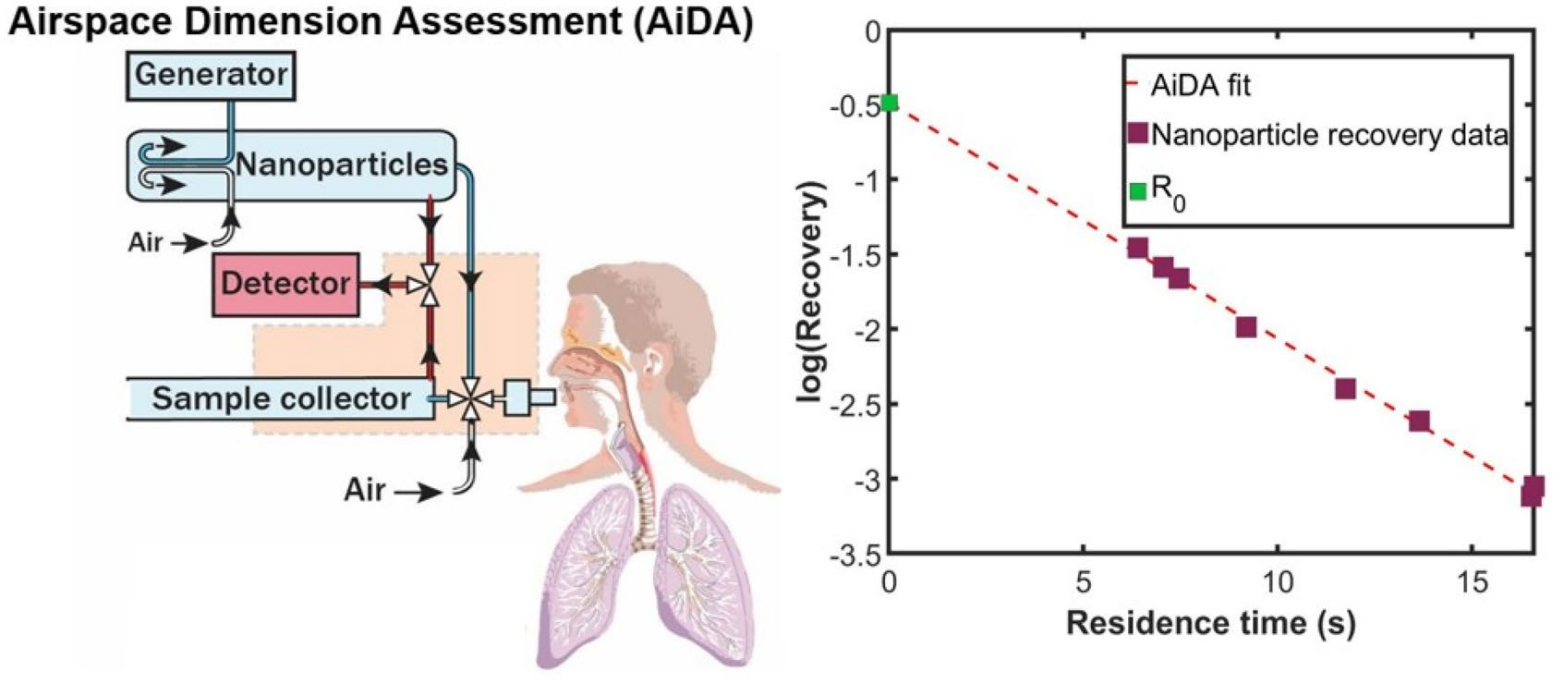
(Left) Schematic illustration of the AiDA method. The aerosol particles from the distal airspaces were sampled to obtain the distal airspace radius *r*_AiDA_ and the zero-second recovery *R*_0._ (Right) Representative recovery data as a function of residence time in the lungs and AiDA fit obtained for one volunteer.

The AiDA method and instrumentation has been described in detail elsewhere^31^. Figure 4 displays a schematic illustration of the AiDA instrument^23^ and example data from one subject.

In short, each subject initially inhaled particle-free air to remove background particles from their lungs. The subject was then instructed to exhale to residual volume (RV) prior to inhaling 50 nm polystyrene latex (PSL) aerosol particles to total lung capacity (TLC), hold their breath for a predefined time and finally exhale. For each measurement, the subject sat upright and a nose clip prevented them from breathing through the nose. Inhaled and exhaled nanoparticle number concentrations were registered for 8 consecutive measurements, with breath-hold times between 5 and 15 s. The inhaled nanoparticle concentration employed for AiDA was less than 10,000 cm^−3^, which is lower than the concentration of ambient nanoparticles in an urban environment^34^.

The probability of particle deposition in the distal airspaces depends on residence time and airspace size. Enlarged airspaces yield a lower deposited fraction, corresponding to a higher particle recovery ($R$) in exhaled gas. The estimation of airspace dimensions using AiDA is based on the solution of the diffusion equation in circular tubes randomly distributed with axisymmetric boundary conditions^21^. The solution shows that the recovery *R* decays exponentially with residence time $\left( t \right)$ in the lung^35^ according to:

$$R\left( t \right) = R_{0} e^{{\frac{\ln \left( 2 \right)}{{ t_{1/2} }}t }} ,$$

where $R_{0}$ is the recovery at zero-second breath-hold and and t_1/2_ is the deposition half-life time. Residence time *t* was established and linear least-squares regression was fitted to the data. From the fit, *R*_0_ and $t_{1/2}$ were determined. *R*_0_ is presumably related to the dynamic phase of breathing and small conducting airways (generation 10–15^36^), but remains to be evaluated further^21,24^. The airspace radius *r*_AiDA_, was calculated from $t_{1/2}$ and the diffusion coefficient ($D$*)* for 50 nm particles according to:

$$r_{{{\text{AiDA}}}} = 2.89\sqrt {Dt_{1/2} }$$

### ^129^Xe DW-MRI measurements

Hyperpolarised ^129^Xe DW-MRI was performed on a GE HDx 1.5T scanner with a flexible transmit/receive quadrature vest coil (Clinical MR Solutions, Brookfield, WI, USA) after the inhalation of a 1 L gas mixture containing 550 mL ^129^Xe (> 25% polarization^37^) and 450 mL N_2_ from the level of function residual capacity. A 3D multiple b-value spoiled gradient echo (SPGR) sequence with compressed sensing was used with a 16 s breath-hold^6^. Specific DW-MRI acquisition parameters were: TE/TR = 14.0/17.3 ms, ^129^Xe diffusion time = 8.5 ms, b = [0, 12, 20, 30 s/cm^2^]. ^129^Xe ADC was calculated using a mono-exponential fit between the signal of the b = 0 (S_0_) and 12 (S_b=12_) s/cm^2^ interleaves:

$$ADC = \ln \left( {\frac{{S_{0} /S_{b = 12} }}{12}} \right).$$

The mean diffusive length scale (*Lm*_*D*_), a measure of mean distal airspace dimension from the SEM was derived by fitting the ^129^Xe diffusion signal from all four b-values to a stretched exponential function^6^:

$$\frac{S\left( b \right)}{{S_{0} }} = \mathop \smallint \limits_{0}^{\infty } p\left( D \right)e^{ - bD} dD = e^{{\left[ { - b \cdot DDC} \right]^{\alpha } }} ,$$

where $p\left( D \right)$ is the probability distribution of different apparent diffusivities within each image voxel, *DDC* is the distributed diffusivity coefficient, and α is the heterogeneity index that describes the deviation from a mono-exponential decay (α = 1). α is thought to be a measure of the underlying complexity or heterogeneity of the geometry of the restricting distal boundaries^38^. From α and *DDC* a numerical expression for $p\left( D \right)$ is estimated^39^. Further, $p\left( D \right)$ is related to diffusion length scales associated with different apparent diffusivities (*D*) by the characteristic diffusion length ($L_{D} = \sqrt {2{\Delta }D} ; {\Delta } =$ diffusion time) which represent a measure of the distribution of microscopic dimensions. *Lm*_*D*_, a measure of mean distal airspace dimensions, is defined as the expectation value of the probability distribution of diffusion length and is related to $p\left( D \right)$ by:

$$Lm_{D} = \mathop \smallint \limits_{0}^{\infty } \sqrt {2\Delta D} p\left( D \right) dD.$$

**Figure 5:**
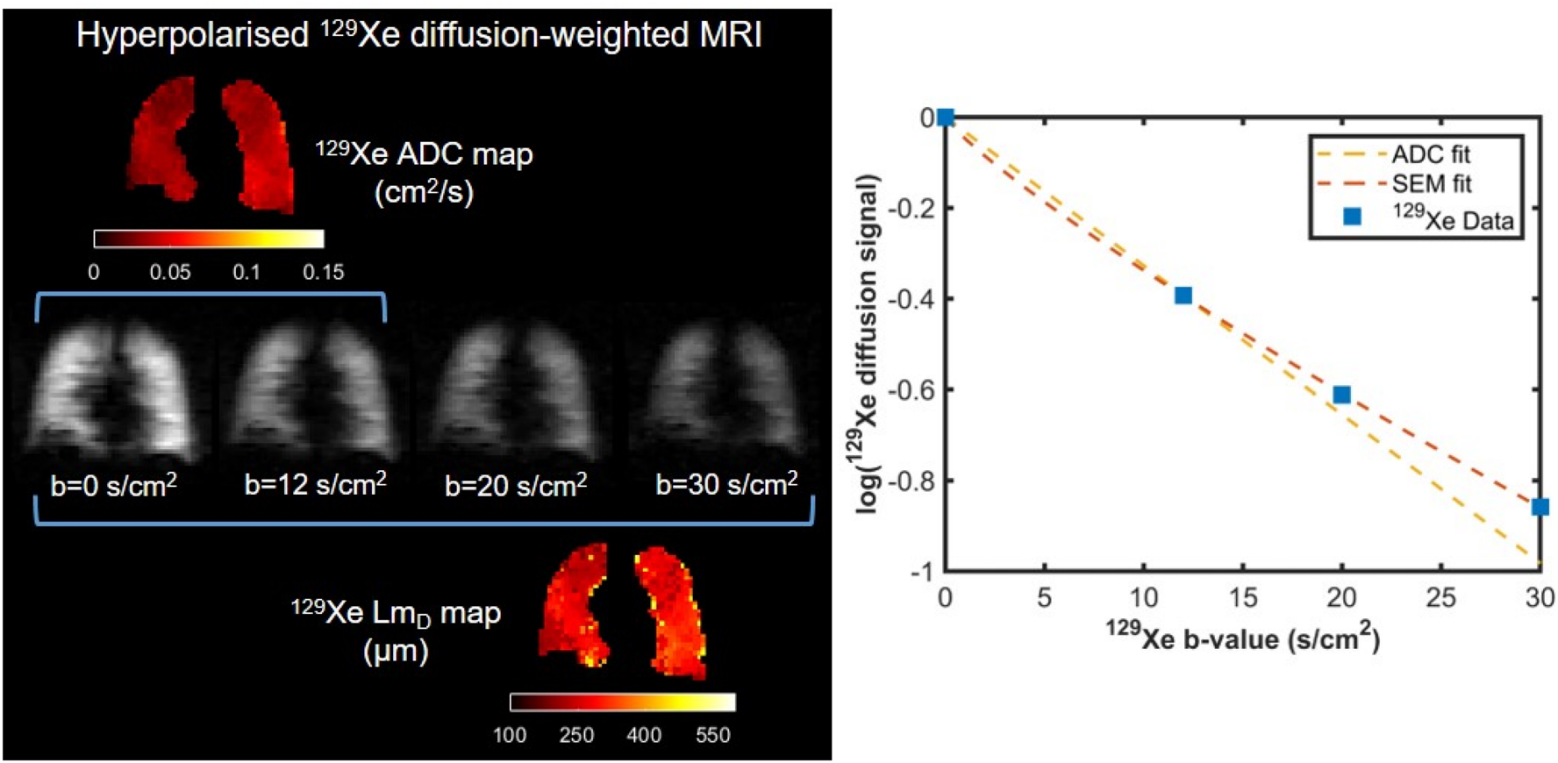
Example hyperpolarised ^129^Xe diffusion-weighted (DW) MRI data from one volunteer. (Left) Maps of ADC and *Lm*_*D*_ values calculated from ^129^Xe DW-MRI. (Right) Representative global ^129^Xe signal as a function of b-value, ADC- and SEM-fits obtained for the same volunteer.

Both ADC and SEM metrics were calculated on a voxel-by-voxel basis, and averaged across the entire lung volume to derive global means. Figure 5 displays examples of ADC and SEM-derived *Lm*_D_ maps from the hyperpolarised ^129^Xe DW-MRI.

### Benchmarking of AiDA versus DW-MRI measurements

All of the AiDA analysis, ^129^Xe DW-MRI lung morphometry calculations and comparisons of metrics were implemented using MATLAB Version 2020a (The MathWorks, Inc., Natick, Massachusetts, United States). Correlations between AiDA variables, ^129^Xe DW-MRI metrics and standard PFT measurements were assessed using Spearman’s rank correlation test. The significance threshold was set at 0.05. Bland–Altman analysis was used to assess the agreement between the *r*_AiDA_ and *Lm*_*D*_.

## Data Availability

The datasets generated during and/or analysed during the current study are available from the corresponding author on reasonable request.
